# Visual evoked potential abnormalities in patients with COVID-19

**DOI:** 10.1590/1806-9282.20231061

**Published:** 2024-03-04

**Authors:** Metin Balduz, Halit Fidancı

**Affiliations:** 1 Adana City Training and Research Hospital, Department of Neurology – Adana, Turkey.; 2 Adana City Training and Research Hospital, Division of Clinical Neurophysiology, Department of Neurology – Adana, Turkey.

**Keywords:** COVID-19, Optic nerve, Evoked potentials

## Abstract

**OBJECTIVE:**

It has been suggested that diseases that may cause visual evoked potential abnormality, such as optic neuritis, may be associated with the coronavirus disease 2019. This study aimed to find out whether there are visual evoked potential abnormalities in coronavirus disease 2019 patients using pattern reversal visual evoked potential and flash visual evoked potential.

**METHODS:**

Patients with a history of coronavirus disease 2019 (coronavirus disease 2019 patients) and controls were included in this prospective case-control study. This study was conducted in the Clinical Neurophysiology Laboratory of Adana City Training and Research Hospital. Individuals without visual impairment were included. Coronavirus disease 2019 patients were required to have clinical features consistent with previous acute infection and a positive nose swab polymerase chain reaction test. Visual evoked potential was applied to coronavirus disease 2019 patients between July 2020 and July 2021. Controls consisted of patients without a history of chronic disease who underwent a visual evoked potential study between June 2017 and June 2018 due to headache or dizziness. Pattern reversal visual evoked potential and flash visual evoked potential were applied to all participants. N75, P100, and N135 waves obtained from pattern reversal visual evoked potential and P1, N1, P2, N2, P3, and N3 waves obtained from flash visual evoked potential were analyzed.

**RESULTS:**

A total of 44 coronavirus disease 2019 patients and 40 controls were included in the study. Age and gender were not different between the two groups. Pattern reversal visual evoked potential parameters were not different between the two groups. Right P2 latency was 114.4±21.1 and 105.5±14.7 ms in coronavirus disease 2019 patients and controls, respectively (p=0.031). Patients with P100 and P2 wave abnormalities were 6 (13.6%) and 13 (29.6%), respectively.

**CONCLUSION:**

This study showed that there may be visual evoked potential abnormalities in coronavirus disease 2019 patients.

## INTRODUCTION

The coronavirus disease 2019 (COVID-19), which emerged in 2019, caused the pandemic. The most common symptoms of COVID-19, caused by severe acute respiratory syndrome coronavirus 2 (SARS-CoV-2), are related to respiratory system such as fever, cough, and dyspnea^1^. However, it has been reported that COVID-19 also affects other systems, including the neurological system^2-4^. Neurological symptoms such as headache, loss of smell, and loss of taste are observed in COVID-19 patients^2-4^. Moreover, it has been reported that neurological diseases such as Guillain-Barré syndrome or cerebrovascular may be associated with COVID-19^3,5,6^. Patients with optic neuritis that may be associated with COVID-19 have also been reported^7-9^. These neurological findings observed in COVID-19 may occur with immune mechanisms or disruption of the blood–brain barrier^5,7,9^. In this case, parts of the peripheral nervous system, such as the optic nerve, or structures related to the central nervous system, such as the visual pathways, may be affected. Visually evoked potentials (VEPs) physiologically reflect visual pathways that extend from the eye to the occipital cortex^10^. VEP can be abnormal in diseases such as optic neuritis. This study aimed to find out whether there are VEP abnormalities in COVID-19 patients using pattern reversal VEP (PVEP) and flash VEP (FVEP).

## METHODS

Controls and patients over 18 years of age with a history of COVID-19 (COVID-19 patients) who applied to the neurology clinic of Adana City Training and Research Hospital (ACTRH) were included in this prospective case-control study. Approval was obtained from the ACTRH ethics committee (No. 77/1342, dated 24 March 2021). Written informed consent was obtained from all patients. VEP was administered to patients who had COVID-19 between July 2020 and July 2021. The interval between the VEP test and the result to be positive for the nose swab polymerase chain reaction (PCR) test for COVID-19 was at least 30 days. Thus, it was aimed to reveal the subacute effects of COVID-19. COVID-19 patients should have (1) fever, cough, dyspnea, or generalized body pain during acute infection and (2) a positive PCR test for COVID-19. As there may be cases of asymptomatic COVID-19 infection, it was decided that the VEP findings of the controls should belong to the dates before the COVID-19 pandemic. Therefore, controls were composed of individuals who underwent VEP between June 2017 and June 2018 for complaints of dizziness or headache. Controls and COVID-19 patients with the following characteristics were not included in the study: (1) neurodegenerative disease, (2) eye diseases such as glaucoma, uveitis, or cataracts, (3) chronic diseases such as diabetes mellitus that may affect the eye, (4) cerebrovascular disease, and (5) abnormalities in the neurological examination. Visual acuity was evaluated using a Snellen chart. The log of the minimum angle of resolution (LogMAR) of all participants should be between 0.00 and 0.20; otherwise, they were excluded from the study.

### Visual evoked potential study

The Cadwell Sierra Summit EMG unit (Cadwell Laboratories, Kennewick, WA, USA) was used for the VEP study. Considering the previously suggested methods, PVEP and FVEP were performed^10^. The recording was made by placing the surface cup electrodes on the Oz and Fz points determined according to the international 10-20 electroencephalography system. VEP study was applied if the impedance of each electrode was <5 kΩ. The band filter frequency was 1–100 Hz. The stimulation frequency was 1 Hz. Sensitivity and sweep speed were set to 2.5 μV/division and 25 ms/division, respectively. VEPs were obtained by averaging 2×200 potentials for each eye. An LED monitor (CBOX 18.5 in.) was used for PVEP. PVEP was obtained using a white-black checkerboard. The contrast difference between checks was 90%. The mean luminance is 240 cd m^–2^. The distance between the monitor and the participant’s eye was 1 m. The dot in the middle of the screen was red. The square size was 52 min of arc. The interval between the stimulation and the checkerboard appeared on the screen was 56 ms due to the use of the LED^11^. N75, P100, and N135 waves were obtained from PVEP. The P100 amplitude calculated by measuring from the N75 peak to the P100 peak was recorded. FVEPs were obtained using Cadwell LED Goggles. The latencies of the N1, P1, N2, P2, N3, and P3 waves of FVEP were included in the analyses. The amplitude of the P2 wave was calculated by measuring from the N2 peak to the P2 peak. We used the P100 wave to identify individuals with PVEP abnormalities. When identifying individuals with FVEP abnormalities, we used the P2 wave, as it is more prominent than other waves and is used in most studies^12,13^. VEP latency was considered abnormal if the VEP latency was delayed more than the reference value derived from VEP findings of controls or more than 10 ms delayed compared with the other side. VEP amplitude was considered abnormal if the amplitude of VEP was less than the reference value or decreased by more than 50% compared with the contralateral side.

### Statistical analysis

Categorical variables were expressed as frequency and percentage, and numerical variables were expressed as mean, standard deviation, median, and interquartile range (25th–75th%). The Mann-Whitney U test was used to compare numerical data between groups. Nominal data were compared between groups using Pearson’s chi-square and Fisher’s exact tests. Reference VEP latencies and amplitudes were obtained from controls. Reference VEP latencies were calculated as mean +2 SD. Due to the variability of the VEP amplitude, the minimum VEP amplitude was considered the reference VEP amplitude. It was decided that at least 40 individuals should be included in the study for each group, with a standard type 1 error rate (0.05) and a power of 0.80^14^. The G Power 3.1 program was used to determine the sample size. It was considered statistically significant if p<0.05. Statistical analyses were performed using the SPSS 22.0 program.

## RESULTS

A total of 52 COVID-19 patients were examined. Two patients (3.8%) had cataracts, four patients (7.6%) had diabetes mellitus, and two patients (3.8%) had visual acuity worse than 0.20. Therefore, 44 COVID-19 (20 males and 24 females) patients and 40 controls (14 males and 26 females) were included in the study. The mean age of COVID-19 patients and controls was 38.2±12.8 (range 18–65) and 35.8±8.6 (range 18–59) years, respectively. Gender and age were not different between the two groups (p=0.330 and p=0.340). The mean (min–max) interval between the application of VEP and the PCR positivity was 47.3±12.1 (30–76) days. The number of patients who had fever, cough, dyspnea, headache, loss of smell, loss of taste, widespread body pain, and diarrhea during acute infection was 17 (39%), 18 (41%), 17 (39%), 27 (61%), 22 (50%), 22 (50%), 26 (59%), and 2 (5%), respectively. Thorax computed tomography findings in 5 (11%) COVID-19 patients were consistent with COVID-19 pneumonia. The symptoms of COVID-19 patients were mild or moderate. There were no patients hospitalized in the intensive care unit. Two of the patients were admitted to the hospital service.

The PVEP and FVEP findings of COVID-19 patients and controls are given in Tables 1 and 2, respectively. At least one P2 or P100 wave could not be obtained in five COVID-19 patients. All controls had P100 and P2 waves. The comparison of PVEP and FVEP values between COVID-19 patients with and without headache, smell, and taste abnormalities is shown in Table 3.

**Table 1.** Comparison of flash visual evoked potential findings between controls and coronavirus disease 2019 patients.

PVEP	Controls mean (SD) (median) (IQR 25th–75th%) (n=40)	COVID-19 patients mean (SD) (median) (IQR 25th–75th%) (n=44)	p-value
Right			
N75 latency (ms)	59.2 (6.14) (58) (52–62)	56.5 (9.4) (56.3) (50–62)	0.110
P100 latency (ms)	90.4 (6.9) (90.5) (85–92)	87.6 (8) (86.6) (82–92)	0.073
N135 latency (ms)	136.2 (14) (137.5) (19–143)	132.8 (15.6) (133.6) (124–142)	0.466
P100 amplitude (uV)	10.1 (3.8) (10) (7–14)	9.7 (4.4) (9.5) (6–13)	0.568
Left			
N75 latency (ms)	57.2 (6.3) (56) (53–62)	54.7 (8.4) (55.5) (48–60)	0.090
P100 latency (ms)	90.7 (6.6) (90.5) (85–95)	88.2 (7.2) (88.7) (84–91)	0.065
N135 latency (ms)	137.1 (14.2) (137.8) (117–142)	131.8 (13.5) (131.3) (121–140)	0.070
P100 amplitude (uV)	10.0 (4.3) (9.5) (7–16)	10.7 (11) (9.8) (6–12)	0.643

IQR: interquartile range; PVEP: pattern reversal visual evoked potential; SD: standard deviation. Right and left PVEP waves could not be obtained in two and three COVID-19 patients, respectively.

**Table 2.** Comparison of flash visual evoked potential findings between controls and coronavirus disease 2019 patients.

FVEP	Controls mean (SD) (median) (IQR 25th–75th%) (n=40)	COVID-19 patients mean (SD) (median) (IQR 25th–75th%) (n=44)	p-value
Right			
N1 latency (ms)	45.9 (9.1) (43) (37–50)	47.7 (11.5) (41.8) (39–60)	0.885
P1 latency (ms)	61 (10.9) (58.5) (51–68)	65.1 (12.8) (61) (53–79)	0.273
N2 latency (ms)	79.8 (11.2) (76.7) (69–87)	83.1 (12.9) (78.6) (71–94)	0.253
P2 latency (ms)	105.5 (14.7) (104.7) (88–116)	114.4 (21.1) (119.1) (94–129)	**0.031**
N3 latency (ms)	121.5 (20.9) (118.5) (103–128)	125.6 (24.4) (112) (106–145)	0.624
P3 latency (ms)	145.2 (21.8) (140.5) (126–155)	149.3 (24.6) (141) (130–172)	0.669
P2 amplitude (uV)	13.2 (6.2) (11.5) (9–17)	15.9 (7.1) (15.8) (10–21)	0.096
Left			
N1 latency (ms)	44.6 (10.3) (42) (37–47)	42 (8.1) (39.7) (37–45)	0.120
P1 latency (ms)	61.3 (10.7) (57.7) (52–66)	59.8 (10.3) (57.8) (53–65)	0.578
N2 latency (ms)	79.4 (10.7) (76.7) (70–90)	81.8 (11.6) (80.9) (72–91)	0.378
P2 latency (ms)	104.3 (13.2) (102.5) (89–116)	110.9 (17.9) (114) (93–126)	0.098
N3 latency (ms)	122.9 (19) (118.5) (103–137)	121.2 (21.9) (113.5) (102–138)	0.541
P3 latency (ms)	145.5 (19.9) (145) (127–152)	145.6 (21.5) (142) (128–160)	0.807
P2 amplitude (uV)	12.7 (5.0) (12.6) (10–16)	16.3 (9.4) (14.7) (9–20)	0.100

FVEP: flash visual evoked potential; IQR: interquartile range; SD: standard deviation. Right N2/P2 and left N2/P2 waves could not be obtained in three and two COVID-19 patients, respectively. The statistically significant value is indicated in bold.

**Table 3.** Comparison of visual evoked potential findings among coronavirus disease 2019 patients with and without headache, smell, and taste abnormalities.

Symptom	PVEP mean (SD) (median)				FVEP mean (SD) (median)			
	Right P100 latency (ms)	Right P100 amplitude (uV)	Left P100 latency (ms)	Left P100 amplitude (uV)	Right P2 latency	Right P2 amplitude (uV)	Left P2 latency (ms)	Left P2 amplitude (uV)
Headache + (n=27)	88.7 (8.4) (89.5)	10 (3.5) (9.5)	89.8 (7.4) (89.5)	9.2 (3.4) (8.9)	114.7 (18.6) (119.1)	16.8 (6.7) (16.6)	110.5 (17.4) (114)	16.9 (9.6) (15.3)
Headache – (n=17)	85.9 (7.5) (83.6)	9.2 (5.5) (8.5)	85.7 (6.4) (85.5)	13.1 (17.1) (10.2)	114 (25.1) (114.9)	14.4 (7.6) (12.3)	111.5 (19.3) (115.1)	15.4 (9.5) (12.8)
p-value	0.356	0.419	0.121	0.904	0.862	0.199	0.698	0.569
Loss of smell + (n=22)	86.2 (6.9) (83.6)	9 (3.5) (9.3)	87.5 (5.1) (86.9)	8.4 (3.2) (9.3)	113.2 (18.4) (120.5)	16 (7.4) (14.7)	108.9 (18.2) (111.1)	14.7 (7.2) (11.9)
Loss of smell – (n=22)	88.4 (9.1) (88.7)	10.3 (5) (10.2)	88.9 (8.9) (89.5)	12.9 (14.8) (10.3)	115.5 (23.7) (117)	15.8 (6.9) (16)	112.9 (17.9) (115)	18 (11.2) (16.6)
p-value	0.529	0.392	0.465	0.167	0.835	0.907	0.414	0.385
Loss of taste + (n=22)	86.7 (6.5) (83.6)	8.7 (3.6) (8.5)	88 (5.2) (87.7)	11.4 (15.3) (8.2)	11.7 (19.6) (119.1)	14.9 (7.2) (13.6)	17.6 (18.9) (111.1)	13.6 (6.9) (11.2)
Loss of taste – (n=22)	88.4 (9.3) (90.3)	10.6 (4.9) (10.9)	88.5 (8.9) (89.5)	10 (4.2) (10.3)	117.2 (22.7) (118.4)	16.9 (7) (16.4)	114.2 (16.7) (119.7)	19.1 (10.9) (18.2)
p-value	0.579	0.170	0.845	0.188	0.523	0.285	0.204	0.059

FVEP: flash visual evoked potential; PVEP: pattern reversal visual evoked potential.

The upper reference limits for right/left P100 and P2 latencies were 104.2/103.9 and 134.9/130.7 ms, respectively. The lower reference limits for right/left P100 and P2 amplitudes were 3.5/4.0 and 4.4/5.0 uV, respectively. Considering the reference VEP values, 6 (13.6%) of the COVID-19 patients had PVEP abnormality and 13 (29.6%) of the COVID-19 patients had FVEP abnormality.

## DISCUSSION

In this study, it was investigated whether VEP abnormalities existed in COVID-19 patients. No abnormalities in VEP waves were found between the two groups, except that right P2 wave latency was found to be more delayed in COVID-19 patients than in controls. However, some COVID-19 patients were found to have VEP abnormalities.

Neurological symptoms and diseases thought to be associated with COVID-19 have been reported. Loss of smell and taste are well-known neurological symptoms of COVID-19^1,2^. Symptoms related to smell or taste abnormalities have been reported at rates ranging from about 30 to 85% in COVID-19, and it was 50% in this study^2,15^. The pathophysiology of the neurological disorders associated with COVID-19 has still not been elucidated, and olfactory spreading is one of the hypotheses^16,17^. As is known, SARS-CoV-2 affects cells via angiotensin-converting enzyme 2 (ACE2) receptors and transmembrane protease serine 2 (TMPRSS2). ACE2 and TMPRSS2 are known to be found in olfactory cells and epithelial support cells^2,16,17^. For this reason, these cells may be affected in COVID-19. This may cause olfactory sensory neurons to be affected indirectly via abnormalities such as ion disturbance, although ACE2 is not present in olfactory sensory neurons^2,16,17^. Similarly, the expression of ACE2 in the pathway extending from the optic nerve to the occipital cortex may explain the VEP abnormalities in the COVID-19 patients involved in this study^18^.

Nervous system involvement in COVID-19 can also be explained by cytokine storm and/or immune response^5,19^. It is reported that COVID-19 may be associated with optic neuritis or GBS, which may indicate that immune events can cause neurological disorders in COVID-19^5,7-9,17,20^. The emergence of these neurological diseases before the full recovery of the COVID-19 disease suggests that the neurological problems are related to the cytokine storm^5,19^. It is difficult to say whether the VEP abnormalities found in COVID-19 patients in this study are due to an immune mechanism or a result of cytokine storm because our study included COVID-19 patients who fully recovered or almost completely recovered and applied to the neurology outpatient clinic at least 30 days after the PCR test was positive.

It has been reported that optic neuritis may be associated with COVID-19^7-9,20^. In an animal model study of the coronavirus, optic neuritis was seen and the central nervous system was affected^21^. In addition, it has been shown in studies on animals that SARS-CoV-2 can cause optic neuritis^22^. Although the cause of optic neuritis associated with COVID-19 is not clearly understood, it can be explained by the presence of ACE2 expression in the optic nerve, which we mentioned earlier^18^. VEP abnormalities in COVID-19 patients in our study may be due to the involvement of the optic nerve. However, the VEP pathway does not only consist of the optic nerve. VEPs reflect a pathway that extends from the eye to the occipital cortex^10^. Findings from this study may indicate that, in addition to the optic nerve, other structures forming the VEP pathway may also be affected by COVID-19.

It has been reported that some viruses may be associated with multiple sclerosis^22^. Viral infections can cause demyelination by directly affecting oligodendrocytes or myelin damage as a result of inflammatory response^23-25^. This may explain the VEP abnormalities found in our study. However, it should be noted that brain magnetic resonance imaging (MRI) scans of the patients are not available.

To the best of our knowledge, there was a recent study conducted with COVID-19 patients and VEP^14^. In that study, PVEP values were not different between healthy individuals and patients with a history of COVID-19, but 12 patients had VEP abnormalities. Similarly, in our study, PVEP values were not significantly different between the two groups. Also, close to our study, we found PVEP abnormality in 6 (13.6%) of the patients. Approximately 30% of COVID-19 patients had P2-wave abnormalities. Unlike PVEP, most of the retina is stimulated with FVEP and a response occurs in a larger area of the cerebral cortex^10^. In COVID-19, larger parts of the retina or areas of the cerebral cortex from which FVEP originates, where PVEP does not originate, may be more affected. However, further studies with VEP and MRI are needed to confirm this hypothesis.

Our study included some limitations. Brain MRIs of the patients were not available. We think that a study to be conducted with VEP and MRI in COVID-19 will be useful to explain the pathophysiology of COVID-19. In addition, it cannot be said that the control group was completely healthy because the patients who applied VEP for disturbances such as dizziness or headache constituted the control group. However, it should be kept in mind that the neurological examinations of the patients were normal and they did not have diseases such as diabetes mellitus that could cause visual impairment. It could also be an advantage if the VEPs of the controls were performed before the COVID-19 pandemic. If controls were taken during the pandemic, there would be a possibility that these controls may have had COVID-19 asymptomatically. This study had some strengths in that the controls were taken before the COVID-19 pandemic. We think that it is important to apply FVEP, which reflects a wider brain region than PVEP, to participants. The finding of VEP abnormalities in some COVID-19 patients may indicate that evoked potentials, such as somatosensory evoked potentials, may be affected by COVID-19. Future studies involving evoked potentials and COVID-19 patients may provide insight into the pathophysiology of COVID-19.

## CONCLUSION

In this study, some VEP abnormalities were found in COVID-19 patients compared with controls. There may be abnormalities in the VEP pathway from the optic nerve to the occipital cortex in COVID-19.
